# Generation and 3-Dimensional Quantitation of Arterial Lesions in Mice Using Optical Projection Tomography

**DOI:** 10.3791/50627

**Published:** 2015-05-26

**Authors:** Nicholas S. Kirkby, Lucinda Low, Junxi Wu, Eileen Miller, Jonathan R. Seckl, Brian R. Walker, David J. Webb, Patrick W. F. Hadoke

**Affiliations:** ^1^University/ BHF Centre for Cardiovascular Science, University of Edinburgh, The Queen's Medical Research Institute

**Keywords:** Medicine, Issue 99, neointima, mouse femoral artery, atherosclerosis, brachiocephalic trunk, optical projection tomography

## Abstract

The generation and analysis of vascular lesions in appropriate animal models is a cornerstone of research into cardiovascular disease, generating important information on the pathogenesis of lesion formation and the action of novel therapies. Use of atherosclerosis-prone mice, surgical methods of lesion induction, and dietary modification has dramatically improved understanding of the mechanisms that contribute to disease development and the potential of new treatments.

Classically, analysis of lesions is performed *ex vivo* using 2-dimensional histological techniques. This article describes application of optical projection tomography (OPT) to 3-dimensional quantitation of arterial lesions. As this technique is non-destructive, it can be used as an adjunct to standard histological and immunohistochemical analyses.

Neointimal lesions were induced by wire-insertion or ligation of the mouse femoral artery whilst atherosclerotic lesions were generated by administration of an atherogenic diet to apoE-deficient mice.

Lesions were examined using OPT imaging of autofluorescent emission followed by complementary histological and immunohistochemical analysis. OPT clearly distinguished lesions from the underlying vascular wall. Lesion size was calculated in 2-dimensional sections using planimetry, enabling calculation of lesion volume and maximal cross-sectional area. Data generated using OPT were consistent with measurements obtained using histology, confirming the accuracy of the technique and its potential as a complement (rather than alternative) to traditional methods of analysis.

This work demonstrates the potential of OPT for imaging atherosclerotic and neointimal lesions. It provides a rapid, much needed *ex vivo* technique for the routine 3-dimensional quantification of vascular remodelling.

**Figure:**
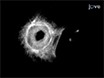


## Introduction

The formation of arterial lesions is central to the high morbidity and mortality associated with cardiovascular disease^1^. Lesion formation is considered to be caused by an unfettered inflammatory response to arterial injury^2^. Atherosclerotic lesions form slowly in response to chronic injury to the arterial wall whereas restenotic lesions develop rapidly following acute mechanical damage (for example, after stent implantation). The mechanisms that contribute to development of arterial lesions have been clarified considerably by the use of appropriate animal models, often in combination with relevant genetic manipulations^1^.

Analysis of lesion size and composition has classically depended heavily on *ex vivo*, 2–dimensional histology (although this is changing with the development of improved methods for *in vivo* and *ex vivo* detection and analysis of lesions in small animals^3^). Histological analysis of arterial lesions is labor intensive, time consuming and provides limited information of 3-dimensional structure. For example, lesion burden is commonly assessed by measuring the cross-sectional area of a lesion (either at randomly selected sites or at the site of maximum occlusion). This provides an incomplete analysis of overall lesion burden. Whole-mount 3-dimensional imaging technology provides a possible solution to this problem but surprisingly few suitable approaches have been described. This may be due predominantly to the size of mouse arteries which are too large for single-photon confocal microscopy but too small for magnetic resonance imaging (MRI)^4^ and X-ray computed tomography (CT)^5^. Application of *ex vivo* MRI and micro CT to the study of atherosclerotic lesions in mice suggests they offer limited resolution, even in relatively large arteries. Added to this, the relatively long acquisition times required limit throughput (and increase scanning costs)^4,6^.

Development of new optical imaging modalities (such as optical coherence tomography^3,7^ and photo-acoustic tomography^8^) offers much potential for improving imaging of lesions in murine arteries. Similar potential is shown by optical projection tomography (OPT) which was developed to allow analysis of mouse embryos. OPT was designed to image specimens ranging from ~0.3-10 mm in diameter^9^. Transmission imaging records the opacity of a semi-translucent sample to polychromatic visible light and, can be used for identification of anatomical structures. Emission imaging records emission of light following excitation at specific wavelengths from endogenous (*e.g.*, collagen, elastin) and exogenous fluorophores in the sample. This can also provide anatomical information (since different tissue components can differ in the type and density of autofluorescent species present). In addition, distribution of immunoreactivity or gene expression can be determined with the use of appropriate fluorescent probes^10^. For either imaging mode (transmission or emission), light is focused to a charge-coupled device to allow iterative image capture as the sample rotates (usually 400 images at 0.9° increments). These can be used for calculation of volume by standard tomographic reconstruction methods (such as filtered back-projection (using a cone algorithm) or iterative reconstruction).

This video demonstrates our novel application of OPT for rapid, quantifiable and cost-effective 3-dimensional analysis of atherosclerotic and neointimal lesions, as previously described in Kirkby *et al.*^11^. The technique was shown to be suitable for quantifying lesion size in three commonly used models: (i) femoral artery wire-injury; (ii) femoral artery ligation, and (iii) diet-induced atherosclerosis in apolipoprotein E deficient (apoE^-/-^) mice.

## Protocol

### 1. Surgical Induction of Neointimal Lesions in the Mouse Femoral Artery

1. Experiments using animals should be performed in accordance with national and institutional ethics requirements. All surgery should be performed using appropriate aseptic technique. Induction of neointimal lesions is achieved using a modification of the technique described by Roque *et al.*^12^ and Sata *et al.*^13^.
2. Weigh male C57Bl6/J mice (Age 10-12 weeks; weight 25-30 g) then anaesthetize by delivering 4-5% isoflurane in an induction chamber. Once anesthesia has been induced, transfer the mouse to a heated mat to maintain body temperature at 37 °C. Continue administration of isoflurane (2-3%) via a mask.
3. Once an appropriate level of anesthesia has been induced (lack of response to toe pinch), provide analgesic cover by administration of buprenorphine (0.1 mg/kg^-1^). Then place the mouse in a supine position and shave the ventral surface of the left hindlimb.
4. Make an incision to expose the muscles of the upper hindlimb and, between the bifurcation with the popliteal artery and the abdominal wall, use blunt dissection to isolate the femoral artery and vein from the femoral nerve. Irrigate the wound as required using 1% w/v lignocaine.
5. Place proximal (close to the abdominal wall) and distal (immediately below the branch with the popliteal artery) temporary ligatures (6/0 Mersilk) around the femoral artery and vein to control blood flow. Then isolate the popliteal artery (for approximately 2-5 mm distal to the branch with the femoral artery) and ligate distally. Place a second, untied ligature beneath the popliteal artery.
6. Make a small incision (arteriotomy) in the popliteal artery, immediately distal to the branch with the femoral artery, preventing bleeding by applying pressure to the proximal temporary ligature. Advance a straight, sprung 0.014” guidewire 1-1.5 cm along the femoral artery in the direction of the abdominal wall and leave in place for 30 sec (**Figure 1A**).
7. Remove the guidewire and ligate the popliteal artery above the arteriotomy, using the ligature placed for that purpose, and taking care not to occlude the femoral artery. NOTE: For ligation-induced injury. Neointimal remodeling without intraluminal injury can be induced by ligating the femoral or popliteal arteries (**Figure 1B** and **1C**). To achieve this follow steps 1.1-1.5. However, do not make the arteriotomy but (avoiding step 1.6) either (i) ligate the popliteal artery immediately distal to the femoral artery or (ii) ligate the common femoral artery at the branch point with the popliteal artery. Then proceed with step 1.8.
8. Remove temporary ligatures, close the wound with a discontinuous external suture (5/0 Mersilk) and apply EMLA cream (2.5% lidocaine, 2.5% prilocaine). Allow the animals to regain consciousness (usually 5-10 min) and ensure they are moving freely around their cage (slight lameness may be evident in the affected leg but this should resolve over the first 2-3 days after surgery) before returning to holding rooms. Mice do not have to be housed singly after surgery.
9. Allow the animals to recover for up to 3 months. Small lesions will begin to appear ~7 days after wire injury and will reach a stable maximum size after ~ 21-28 days.

### 2. Induction of Atherosclerotic Lesions in the Apolipoprotein E^-/-^ Mice

1. Administer western diet (0.2% cholesterol; Research Diets, USA) to male, 6 week old ApoE-null mice (bred in house) for 12 weeks.
2. Atherosclerotic lesions are often visible on gross inspection of the aortic arch and its major branches (**Figure 2**).

### 3. Analysing Arterial Lesions using Optical Projection Tomography (OPT)

NOTE: OPT images of lesions in murine femoral arteries and aortic arch samples were obtained using an optical projection tomograph.

1. Euthanize mice by transcardiac perfusion fixation and exsanguination under terminal anesthesia (80 mg/kg sodium pentobarbital), using heparinised (10 U/ml) phosphate buffered saline (PBS) followed by 10% neutral buffered formalin.
2. Isolate femoral arteries or the aortic arch and its major branches (left carotid artery, left subclavian artery, brachiocephalic trunk), as appropriate, and remove extraneous peri-adventitial material. Post-fix in 10% buffered formalin O/N, before storage in 70% ethanol until needed.
3. Embed arteries in 1.5% low melting point agarose, pre-filtered through Whatman 113 V paper. Attach each sample to a magnetic OPT mount with cyanoacrylate adhesive with the vessel axis in line with that of the mount. Trim excess agarose to a conical shape. Dehydrate in 100% methanol for at least 12 hr.
4. Clear vessels by immersion (for 12-24 hr) in a mixture of benzyl alcohol and benzyl benzoate (1:2 v/v).
5. Place cleared samples in a calibrated tomograph. Set resolution to 1,024 x 1,024, and determine an optical magnification that allows the entire area of interest to be seen. OPT volume is isotropic the z-axis is reconstructed to the same resolution (*i.e.*, 1,024 x 1,024 x 1,024), voxel size ~200 µm. This may represent an over-estimate of resolution as there are likely to be reconstruction artifacts. Adjust sample position so that it rotates upon its own axis in the center of the field of view in the bright-field, transmission channel.
6. In the GFP1 filter emission channel (excitation filter 425 nm with 40 nm band-pass; emission filter: 475 nm long pass), focus the specimen and adjust exposure time to maximize the dynamic range of the resulting image (avoid over-saturation). Scan the vessel in the GFP1 emission channel only, with a 0.9°rotation step.
7. At completion, confirm quality of data acquisition using DataViewer software. Remove specimen from the scanner.
8. To allow subsequent histological analysis, place sample in 100% methanol for >24 hr before processing to paraffin wax as normal.

### 4. Image Reconstruction and Analysis

Tomographic re-construction by filtered back-projection is performed using NRecon software or similar. Reconstructions can be performed unattended, in batches.

1. Improve image quality by compensating for misalignment and adjusting image intensity levels.
2. Check the quality of image reconstruction using DataViewer software.
3. Identify the appropriate section of the sample for analysis. Keep this length consistent between vessels if luminal dimensions are to be recorded.
4. Define the outline of the lesions by manually tracing the appropriate border for 1 in every 50 re-constructed cross-sections.
5. Check each interleaved cross-section to ensure computer-generated interpolations are correct. Manually adjust the border where necessary.
6. Set the grey-level threshold so that only the lesion is selected and export the measurement data.
7. For each scan, define a vertical region of interest containing the lesion and trace the border between media and neointima (*i.e*., the position of the internal elastic lamina) for every 50^th^ scan-line. Interpolate intima/media borders for the interleaved scan lines in software, and verify and correct the fit where required. Further segment this defined three dimensional volume to a manually-defined intensity threshold to produce a binary image set in which white pixels represent neointima and black pixels represent patent lumen.
8. Measurements taken include: total lesion volume (object volume), luminal volume (total volume – object volume) and the distribution of lesion and lumen cross-sectional area along the axial length of the studied vessel.

## Representative Results

Preliminary scanning of healthy (unlesioned) murine femoral arteries (n = 5) demonstrated that transmission imaging did not provide useful images. This was a consequence of the cleared arteries becoming too transparent (rather than too opaque) for transmission imaging.However, this is beneficial for emission imaging as there is no absorbance/scattering of the emitted signal . In contrast, femoral arteries autofluoresce strongly in the emission channel, with the greatest signal following excitation at 405–445 nm (consistent with a 410 nm excitation peak for elastin^14^). 2-dimensional slices reconstructed from these images clearly distinguished the media from the lumen and adventitia and lumen.

In murine femoral arteries harvested 28 days after wire- (n = 6) or ligation- (n = 5) induced injury neointimal thickening was evident in non-tomographic emission projections (**Figure 3A**). In reconstructed 2-dimensional slices, concentric neointimal lesions could be distinguished from the media by their weaker emission (**Figure 3B** and **Figure S1**).

OPT emission images of whole mount samples of the aortic arch and its major branches from atherosclerotic mice (n = 8) identified lesions with the anticipated anatomical distribution (*i.e*., in the lesser curvature of the aortic arch, the brachiocephalic artery, and the origins of the left carotid and left subclavian arteries (**Figure 4A**). Cross-sectional images indicated that these were typically eccentric lesions and were readily distinguished from the media and lumen (**Figure 4B**,** Figures S2** and **S3**).

Processing arteries for histological analysis following OPT confirmed the non-destructive nature of OPT, with sections successfully stained using histological (United States Trichrome, Picrosirius red) and immunohistochemical (α-SMA, Mac-2) techniques (**Figures 3C** and **4C**).

Measurement of lesion size using OPT has been shown to be consistent with measurements obtained using image analysis of histological sections taken from the same artery^11^.

Planimetric measurements of lesion area obtained by OPT and histology correlated closely by linear regression for wire- (R^2 ^= 0.92) and ligation-induced (R^2^ = 0.89) neointimal lesions and atherosclerotic plaques (R^2^ = 0.85). An important benefit of OPT is its ability to enable 3-dimensional analysis. By developing volumetric quantification of lesions with this technique, we were able to record lesion volumes in wire- (0.1100 ± 0.0091 mm^3^; n = 6) and ligation-injured femoral arteries (0.0200 ± 0.0089 mm^3^; n = 5) and also in atherosclerotic brachiocephalic arteries (0.180 ± 0.018 mm^3^; n = 8). Measurements were highly reproducible (coefficients of variation 5.4%, 11.4% and 4.8%, respectively, n = 4) for all types of lesion. Neointimal lesions in wire-injured vessels were larger (p <0.0001) than those produced by ligation, consistent with the greater degree of damage inflicted by the former.

The data generated could also be expressed as lesion profiles (**Figure 5**) and rendered for dynamic, qualitative evaluation (see **Figures S1**-**S3**). This approach clearly demonstrated the extent of lesion formation in response to different injury procedures and highlighted the uneven distribution of lesion formation in injured vessels.


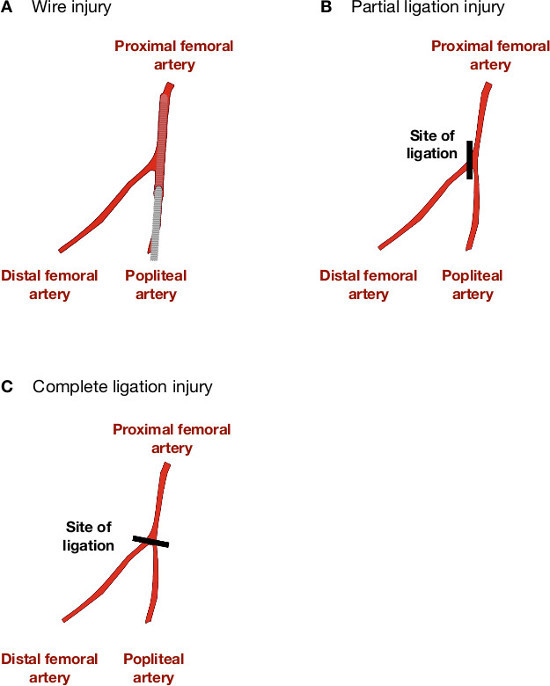
**Figure 1:Methods for initiating lesion formation in murine femoral artery. **(**A**) Retrograde insertion of an angioplasty guidewire into the femoral artery, by means of an arteriotomy in the popliteal artery stimulates lesion formation in response to stretch injury and removal of the endothelium. Blood flow is re-established over the injured section of vessel. (**B**) Neointimal proliferation in the absence of intraluminal stretch, denudation or interruption to blood flow can be induced by ligating *either* the femoral or the popliteal artery immediately distal to the femoral artery bifurcation. (**C**) A more severe non-denuding injury/ proliferation response can be induced by ligating both the femoral and popliteal arteries across the branch point of the common femoral artery. This technique will also block blood flow in the distal portion of the femoral artery.


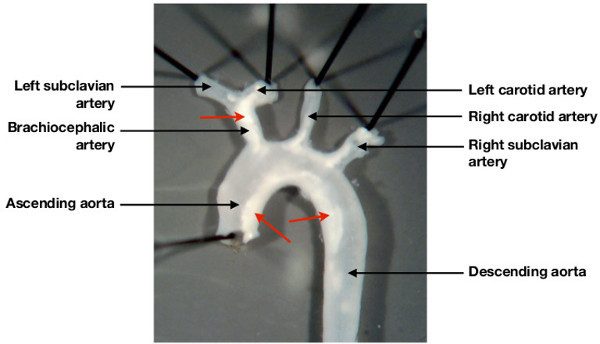
**Figure 2: Characteristic deposition of atheroma in the mouse aortic arch. **Atherosclerosis prone (Apolipopotein E deficient mice) fed a high cholesterol western diet for 12 weeks develop a characteristic pattern of lesion deposition in the aortic arch and its major branches. As demonstrated, lesions are visible (arrows), by gross inspection under a dissecting microscope, in the aortic arch, the brachiocephalic artery, and in the ostia of the left carotid artery and left subclavian artery. 


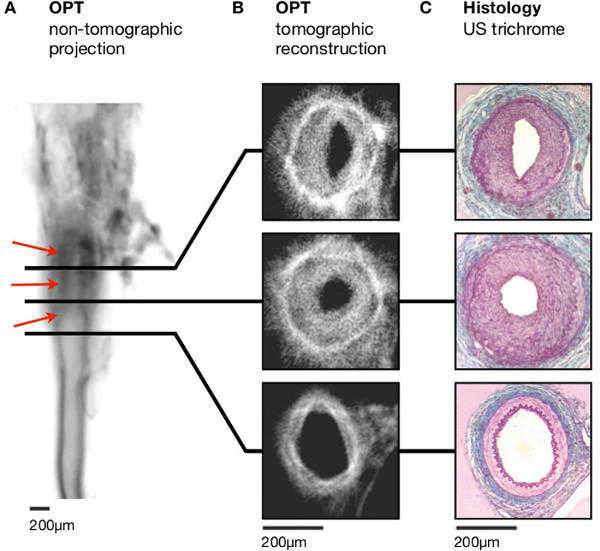
**Figure 3: Lesion formation following ligation of the left femoral artery. **(**A**) Non-tomographic fluorescence emission images (inverted to increase clarity – dark regions correspond to stronger emission) allow identification of intimal thickening (red arrowheads). (**B**) Distinct vascular regions and the lumen can be distinguished in tomographic reconstructions. (**C**) Histological analysis (United States trichrome) emphasises the clear resemblance with images obtained using OPT. Scale bars in (A-C) are 200 mm. Adapted from Kirkby *et al*.^11^ Scale bars in (A-C) are 200 µm.


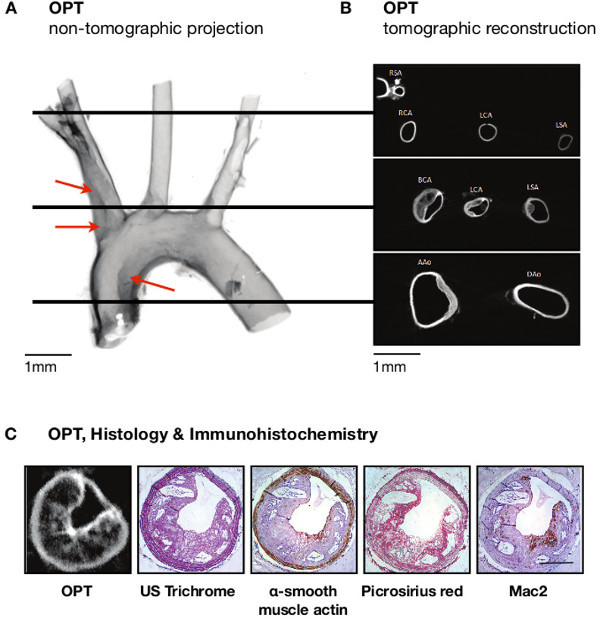
**Figure 4:Imaging of atheroma in the aortic arch of atherosclerosis prone mice.** (**A**) Atheroma (red arrowheads) is readily apparent in non-tomographic images (inverted so that darker regions indicate stronger emission, thus improving clarity) of the aortic arch, in sites predicted as atheroma-bearing by inspection under light microscopy (see Figure 2). (**B**) This pattern of distribution is confirmed in tomographic cross-sections. (**C**) Histological (United States trichrome) staining shows close similarity with tomographic sections, and immunohistochemistry using several different antibodies emphasises the complementary nature of OPT with traditional approaches to lesion analysis. Scale bars in (A–B) are 1 mm; Scale bar in (C) is 250 µm. RSA, right subclavian artery; RCA, right carotid artery; LCA, left carotid artery; LSA, left subclavian artery; BCA, brachiocephalic artery; AAo, ascending aorta; DAo, descending aorta. Adapted from Kirkby *et al*.^11^


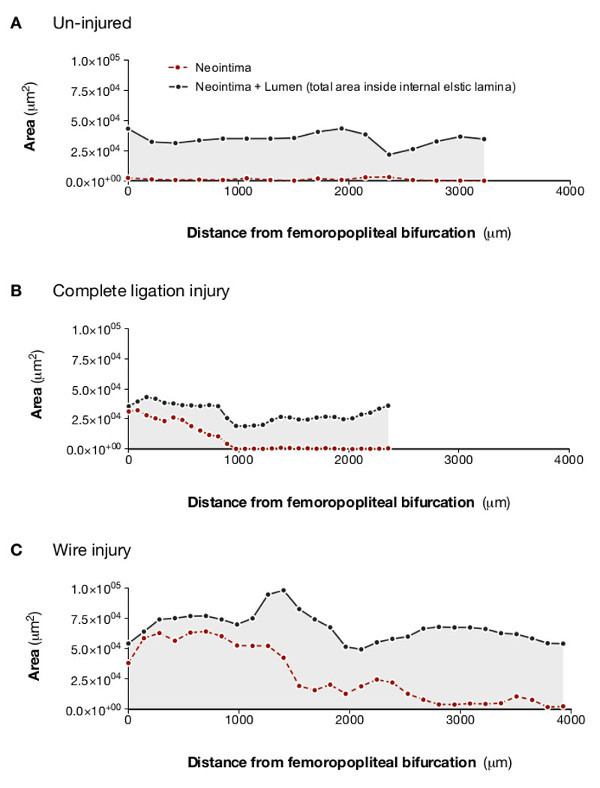
**Figure 5:Analysis of lesion and lumen profiles indicates varying extent of neointimal proliferation in response to different methods of arterial injury. **Optical projection tomography allows lesion and lumen cross sectional measurements to be plotted against distance along the femoral artery. This clearly demonstrates that, compared with an uninjured artery (**A**), partial ligation (**B**) produces small, relatively discrete lesions, whereas total ligation (**C**) produces complete occlusion at the site of ligation but the lesion does not extend far along the artery. Intraluminal wire injury (D) produces a lesion that almost completely occludes the distal portion of the sample and extends along the entire length of the scanned section of artery. Adapted from Kirkby *et al*.^11^

**Figure S1.** **Animated reconstruction of cross-sectional images obtained from a mouse femoral artery following ligation injury.**This type of animated image is useful for both qualitative and quantitative analysis. As the animation moves from the proximal to the distal sections of the artery the gradual development of an occlusive neointima, discernible from the lumen and the media, is readily apparent. Side branches can be easily identified and there is evident luminal occlusion and outward remodelling of the artery as the lesion increases in size. Complete occlusion of the vessel occurs once the site of ligation is reached. Adapted from Kirkby *et al*.^11^

**Figure S2.** **Animated reconstruction of cross-sectional images of an aortic arch from an atherosclerosis-prone mouse. **The animation commences with cross-sections of the ascending (left – which appears first) and descending (right) aorta. Small lesions appear in the ascending aorta as the scan moves in the direction of the aortic arch. The images then move through the arch to show the heavily lesioned ostia of the brachiocephalic (left), left carotid (middle) and left subclavian (right) arteries. As the scan moves distally along these branches the lesions gradually reduce and disappear, first in the subclavian artery, then in the carotid and finally in the brachiocephalic artery. Interestingly, the lesion in the brachiocephalic artery moves onto the flow divider as this vessel divides into the right carotid and right subclavian arteries. Adapted from Kirkby *et al*.^11^

**Figure S3.** **Animated, volume-rendered image of an aortic arch from an atherosclerosis-prone mouse. **Optical projection tomography allows generation of 3-dimensional images, in this case demonstrating lesion distribution in the aortic arch of an apolipoprotein E deficient mouse. (**A**) Atheroma is present in the expected sites (throughout the brachiocephalic artery, in the ostia of the left carotid and subclavian arteries and in the lesser curvature of the aortic arch). (**B**) Segmentation and rendering of lesion (shown in red) bearing cross-sections emphasises the distribution of plaques when superimposed on the original image. Adapted from Kirkby *et al*.^11^

## Discussion

3-dimensional analysis has great potential for replacing or adding to the 2-dimensional histological techniques that still underpin the majority of investigations of arterial lesion formation. Here OPT is shown in small murine arteries (with murine femoral arteries probably representing the smallest vessels that can be analyzed successfully using this technique). It is, however, also suitable for use with arteries (and lesions) from other species, including small-to-medium sized human vessels; our group has successfully used the technique to analyze lesions in rabbit aorta (Bezuidenhout *et al.*; unpublished). OPT promises faster analysis and increased structural information compared with traditional histology and has the advantage of not preventing subsequent analysis of the sample using both histological and immunohistochemical techniques.

The images produced using OPT gave anatomical detail, showing sites of lesion formation and the size of lesions in these areas. The arteries used in these investigations are probably close to the limit of resolution for the technique and image quality is therefore impaired to a certain degree by artifacts (probably resulting from rotation misalignment, incomplete clearing, reflection/refraction at the agarose vertices and focusing problems). Despite this, the details required (*i.e.*, layers of the vessel wall) remain discernable and therefore the technique is extremely useful for the quantification of individual layers. Indeed, images could be quantified rapidly and reproducibly to provide measurements of lesion and luminal volume in plaque-bearing sections of the vessel, as well as cross-sectional areas of the lesion and lumen at selected sites in the sample. Large (aorta) and medium sized (femoral, carotid, subclavian) murine arteries – those generally used for analysis of atherosclerotic and neotintimal lesion formation in mice – were successfully analyzed using this method. Indeed we have now used OPT to demonstrate the effect of pharmacological interventions and genetic manipulation on atherosclerotic and neointimal lesion size. For example, endothelin receptor blockade altered neointimal lesion formation whereas selective deletion of the endothelin B receptor from the vascular endothelium did not^15^. In atherosclerosis prone mice, genetic deletion of the enzymes 11β-HSD1^16^ or galectin 3^17^ were shown to reduce the size of atherosclerotic lesions.

Quantification of lesion volume is an obvious benefit of OPT. It gives a more informative indication of the total lesion burden in an artery^4^ than is usually obtained with histological methods. Analyzing the entire lesion reduces selection bias and error that will inevitably occur when discrete sections of a vessel are chosen for analysis. Production of longitudinal lesion profiles is a further strength of OPT, emphasized by the comparison of lesions induced by different types of injury^13,16^ (**Figure 5**). For example, both complete ligation and wire-insertion induced almost total occlusion close to the femero-popliteal bifurcation. Wire injury, however, produced lesions that extended along the entire length of the scanned section, whereas lesions induced by arterial ligation rapidly diminished in size and disappear. This pattern is consistent with the greater extent of injury caused by insertion of the angioplasty guidewire. Generating similar results using histological sections is expensive, time consuming and labor intensive.

The advantages of OPT include the quality of the images it produces and its relative speed and simplicity (we have routinely scanned 20 vessels per day). Image quality appears superior, or at least comparable, to other methods for generating 3-dimensional images *ex vivo* (such as MRI and micro- CT), yet OPT requires shorter scan times (integration time for our studies was typically 1-2sec/image) and is less expensive. Sample preparation extends over several days but requires little labor, vessels can be prepared in batches, and data can be acquired in one session. Consequently, throughput is high and does not require extended use of the scanner. Importantly, the non-destructive nature of OPT means it can be used to identify sites of interest for immunohistochemical examination; thus reducing the amount of cutting and staining required. It is possible that development of high resolution ultrasound will provide an alternative method for volumetric quantification of lesions in arteries this size, but the authors are unaware of any publications that demonstrate this application.

Perhaps unsurprisingly, image quality in OPT is inferior to microscopic techniques (which can, of course, only be performed on smaller samples). Proposed refinements to reconstruction of data may address this limitation by allowing future improvement of image quality^19,20^. Another methodological concern is that tissue processing alters characteristics of the sample. For example the lipophilic nature of the clearing agent, benzyl alcohol/benzyl benzoate (BABB), is likely to remove lipid from atherosclerotic lesions, whilst prior dehydration may cause shrinkage (although, of course, dehydration and lipid removal steps are also a feature of sample preparation for embedding in paraffin wax). BABB was used in this investigation as, in comparison with hydrophilic clearing agents (*e.g.* glycerol^21^) it causes only small changes in morphology.

There are several possibilities for further development and refinement of OPT, particularly with regard to tracking the 3-dimensional arrangement of key cells and signaling factors involved in arterial remodeling. The strong autofluorescence of arterial tissue, which is such an advantage in the generation of anatomical images, is not quenched by existing methods of bleaching^22^ and may restrict the use of fluorescent probes to assess RNA and protein distribution patterns. The use of colorimetric probes (*e.g.* β-galactosidase) visualized by transmission imaging may overcome this limitation.

To conclude, OPT has great potential for 3-dimensional imaging of lesions in the intima of murine arteries. It represents a considerable advance on 2-dimensional methods which are generally labor intensive and do not effectively represent total lesion volume. OPT is relatively fast, convenient and non-destructive. New developments in image analysis promise to further increase the power and utility of the technique.

## Disclosures

The authors have no competing financial interest.
